# Monogenic Defects of Beta Cell Function: From Clinical Suspicion to Genetic Diagnosis and Management of Rare Types of Diabetes

**DOI:** 10.3390/ijms251910501

**Published:** 2024-09-29

**Authors:** Anastasios Serbis, Evanthia Kantza, Ekaterini Siomou, Assimina Galli-Tsinopoulou, Christina Kanaka-Gantenbein, Stelios Tigas

**Affiliations:** 1 Department of Pediatrics, University of Ioannina, 45110 Ioannina, Greece; kantzaevina@gmail.com (E.K.); eksiomou@uoi.gr (E.S.); 2 Department of Endocrinology & Diabetes Center, University of Ioannina, 45110 Ioannina, Greece; stigas@uoi.gr; 3 2nd Department of Pediatrics, School of Medicine, Faculty of Health Sciences, Aristotle University of Thessaloniki, AHEPA University General Hospital, 54636 Thessaloniki, Greece; agalli@auth.gr; 4 Division of Endocrinology, Diabetes and Metabolism and Aghia Sophia ENDO-ERN Center for Rare Pediatric Endocrine Disorders, First Department of Pediatrics, Medical School, Aghia Sophia Children’s Hospital, National and Kapodistrian University of Athens, 11527 Athens, Greece; chriskan@med.uoa.gr

**Keywords:** monogenic diabetes, neonatal diabetes, MODY, syndromic diabetes, mitochondrial diabetes

## Abstract

Monogenic defects of beta cell function refer to a group of rare disorders that are characterized by early-onset diabetes mellitus due to a single gene mutation affecting insulin secretion. It accounts for up to 5% of all pediatric diabetes cases and includes transient or permanent neonatal diabetes, maturity-onset diabetes of the young (MODY), and various syndromes associated with diabetes. Causative mutations have been identified in genes regulating the development or function of the pancreatic beta cells responsible for normal insulin production and/or release. To date, more than 40 monogenic diabetes subtypes have been described, with those caused by mutations in *HNF1A* and *GCK* genes being the most prevalent. Despite being caused by a single gene mutation, each type of monogenic diabetes, especially MODY, can appear with various clinical phenotypes, even among members of the same family. This clinical heterogeneity, its rarity, and the fact that it shares some features with more common types of diabetes, can make the clinical diagnosis of monogenic diabetes rather challenging. Indeed, several cases of MODY or syndromic diabetes are accurately diagnosed in adulthood, after having been mislabeled as type 1 or type 2 diabetes. The recent widespread use of more reliable sequencing techniques has improved monogenic diabetes diagnosis, which is important to guide appropriate treatment and genetic counselling. The current review aims to summarize the latest knowledge on the clinical presentation, genetic confirmation, and therapeutic approach of the various forms of monogenic defects of beta cell function, using three imaginary clinical scenarios and highlighting clinical and laboratory features that can guide the clinician in reaching the correct diagnosis.

## 1. Introduction

The concept of a single genetic abnormality leading to diabetes emerged decades ago by clinicians who observed hyperglycemia and diabetes presenting early in life in neonates or infants, as well as in several family members diagnosed with diabetes in adolescence or early adulthood and following a mendelian pattern of inheritance [1]. These clinical observations corresponded to what was later described as neonatal diabetes mellitus (NDM) and maturity-onset diabetes of the young (MODY), respectively. These two clinical entities together with some forms of syndromic diabetes are collectively known as monogenic defects of beta cell function [2].

In the last three decades, several genetic defects have been identified as causes of the various subtypes of monogenic diabetes. MODY subtypes were initially named after the order by which the respective causative locus and gene were described in the literature. Thus, “MODY1” was used for the subtype involving the *HNF4A* gene, “MODY2” for the one caused by mutations in the *GCK* gene, and so forth up to MODY14, the latest subtype identified (Table 1) [3].

Gradually, it became evident that the various types of monogenic diabetes share some common features, and, moreover, that monogenic diabetes overlaps not only phenotypically but also genetically with type 1 (T1D) and type 2 diabetes (T2D). As an example, depending on the severity of a single gene’s mutation, an individual may present with NDM, with a type of MODY, or with T2D later in life [3,4]. These observations, together with an improved understanding of the genetic architecture of monogenic diabetes, led to a gradual transition in the classification and nomenclature of the various subtypes, from a clinically based system to one based on molecular genetics. As an example, the preferred term for MODY2 is GCK-MODY (glucokinase-MODY), while the preferred term for NDM caused by mutations in the *KCNJ11* gene is KCNJ11-NDM. In addition, to better characterize the various subtypes of monogenic diabetes, it has been suggested that the way the disease is transmitted is also included in each specific name. For example, MODY caused by a heterozygous dominant mutation in the *GCK* gene can be called “dominant GCK-diabetes”, while homozygous recessive mutations in the same gene leading to NDM is called “recessive GCK-NDM” [3].

The evolution in the understanding of the genetic causes of monogenic diabetes has increased the accuracy of our diagnoses, has improved our ability to foresee the possible clinical course, and has contributed to selecting the best treatment for any given patient. Despite these advances, identification of monogenic diabetes cases is still based on referral of suspicious cases for genetic testing by individual physicians. Therefore, clinical suspicion based on signs, clinical findings, and laboratory tests is crucial in the diagnostic process of such patients, since systematic universal screening for monogenic diabetes has not yet been established [5]. In this regard, the current review aims to summarize the latest knowledge on clinical presentation, genetic testing, and therapeutic approach of the various forms of monogenic defects of beta cell function, using as examples three imaginary clinical scenarios and highlighting clinical and laboratory features that can guide the physician in ultimately reaching the correct diagnosis.

## 2. Methods

A literature search was conducted on the PubMed/Medline database for studies from peer-reviewed journals published between 1 January 1980 and 31 May 2024, to identify relevant papers using the following keywords: “monogenic diabetes”, “neonatal diabetes”, “MODY”, “syndromic diabetes”, “mitochondrial diabetes”, “monogenic beta cell defects”, “clinical features”, “diagnosis”, and “treatment”. Only studies in English were included, and case series, cross-sectional studies, observational studies, and reviews were all included in the initial evaluation. Relevance was evaluated according to title and abstract, when available. Full-text articles of all relevant studies were retrieved and reviewed. Any relevant papers identified through a manual search of the references from the retrieved articles were also included. Results are presented in a narrative way, using examples of an imaginary case scenario typical of each of the three main categories of monogenic defects in beta cell function, namely neonatal diabetes, MODY, and syndromic diabetes.

## 3. Discussion

**Figure 1:**
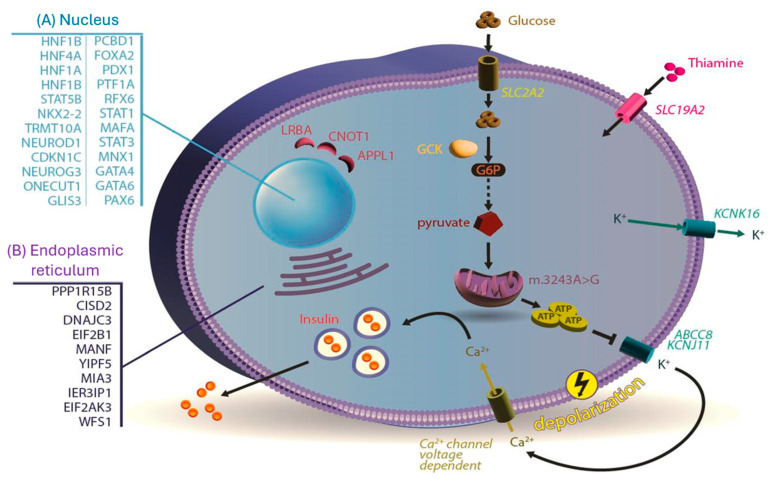
The protein products of the genes involved in the various forms of monogenic diabetes are presented. Several of the β-cell organelles are involved, including the nucleus, endoplasmic reticulum, cytosol, mitochondria, and plasma membrane. In the nucleus, genes responsible for transcription factors vital to pancreatic islet development and beta cell function and maintenance (e.g., *HNF1A*, *HNF4A*, *HNF1B*, *PDX1*, *PTF1A*, and *GATA6*) are involved in various monogenic diabetes forms (**A**). Mutations in other genes (e.g., *WFS1*, *MIA3*, *CISD2*, *MANF*, *YIPF5*, *EIF2AK3*, and *DNAJC3*) are associated with monogenic diabetes by affecting endoplasmic reticulum calcium homeostasis (**B**). ATP-sensitive potassium (K_ATP_) channel dysfunction can cause monogenic diabetes due to mutations in *ABCC8*, *KCNJ11*, and *KCNK16* genes, along with other ion transporters (e.g., *SLC2A2* and *SLC19A2*) of the plasma membrane. Interestingly, no Ca-channel mutations have been described as a cause of monogenic diabetes. ATP: adenosine triphosphate, Ca: calcium, G6P, glucose-6-phosphate, GCK: glucokinase, and K: potassium.

The various monogenic defects of beta cell function and the specific mutations in genes involved in pancreatic beta cell development and/or function leading to insufficient insulin production, and thus hyperglycemia and diabetes, are shown in Figure 1. As already mentioned, this group of diseases includes the rare NDM that appears during the first six months of life, the more frequently diagnosed MODY, and rare syndromic types of diabetes. Each of these three groups of diseases are presented below after a typical case scenario. Other subtypes of monogenic diabetes include congenital severe insulin resistance syndromes [6] and generalized and partial lipodystrophy [7], which are beyond the scope of this review.

### 3.1. Monogenic Neonatal Diabetes Mellitus

#### 3.1.1. Case Scenario 1

A 10-week-old male infant presented with 6 h of persistent vomiting. He had been breastfed since birth but has had difficulty maintaining his weight despite supplementation with a commercial formula. Over the past 24 h, he had been nursing poorly and became restless. On physical examination, his temperature was 37.1 °C, heart and respiratory rate were 180 beats/min and 32 breaths/min, respectively, blood pressure was 74/48 mm Hg, and body weight was 3100 g (with a birth weight of 2300 g). He responded to tactile and verbal stimuli but had labored breathing. He had no dysmorphic features, acanthosis nigricans, or focal signs of infection. He appeared dehydrated with signs of shock. Initial laboratory tests revealed the following: sodium levels of 124 mEq/L [normal range (NR): 135–145], potassium levels of 3.4 mEq/L (NR: 3.5–5.5), glucose levels of 783 mg/dL (NR: 70–99), and blood ketone levels of 6.6 mmol/L (NR < 0.5). His venous blood gas analysis showed a pH of 7.18 (NR: 7.35–7.45), Pco_2_ of 24 mm Hg (NR: 35–45), and bicarbonate of 13 mEq/L (NR: 22–26). Rehydration with normal saline was administered intravenously, and after admission to the pediatric care unit, an insulin infusion was started. The infant’s condition improved over the next 24 h with resolution of the acidosis, ketosis, and hyperglycemia. After initiation of breastfeeding, his glucose levels increased again into the 300 mg/dL-range. 

The infant described in imaginary case scenario 1 has several clinical features consistent with NDM. More specifically, he was born small for gestational age, had failure to thrive in the first postnatal weeks, and presented with diabetic ketoacidosis (DKA) before the age of 6 months.

#### 3.1.2. Neonatal Diabetes Mellitus

NDM is a very rare diabetes form with a reported incidence ranging between 1 in 90,000 and 1 in 160,000 live births, with most cases being heterozygous [8]. It is caused by more than 30 different single gene mutations, up to 80% of which are de novo mutations affecting pancreatic development or beta cell function and is characterized by hyperglycemia of varying severity and duration. It can appear as early as during the neonatal period (first 28 days) or as late as the second infantile period (up to 12 months), but it usually appears between the first and sixth month of life [9]. In contrast, autoimmune T1D is highly unlikely to present as early during infancy.

**Figure 2:**
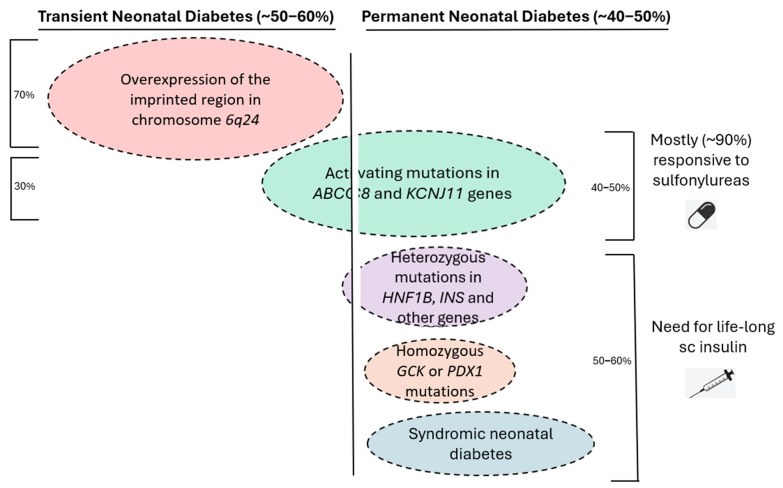
Schematic representation of the association between specific genetic causes and transient or permanent neonatal diabetes mellitus (NDM), as well as treatment requirements for permanent NDM cases. The size of the ovals roughly approximates relative frequencies. With data from [3,10,23].

Depending on the specific genetic cause, NDM can be either transient or permanent (Figure 2). Transient NDM represents about 50–60% of all cases and is usually caused by overexpression of the imprinted region in chromosome *6q24* (two thirds of the cases) or by *KCNJ11* or *ABCC8* activating mutations [10,11]. Isolated case reports of transient NDM due to mutations in the *INS* gene have also been reported [12]. Transient NDM is characterized by resolution of the hyperglycemia usually by the 12th week of life and definitely by the 18th month, with the possibility of recurrence later in life, usually during adolescence or young adulthood [9,13,14]. In such cases, it is important to confirm the diagnosis of NDM, since these patients will often respond to oral antidiabetic medications without the need for insulin therapy [15]. On the other hand, permanent NDM cases have been mostly linked to specific activating mutations in *ABCC8* and *KCNJ11* genes which encode for the two subunits SUR1 and Kir6.2 of the ATP-sensitive potassium (K_ATP_) channel of the beta cell, respectively [16]. Milder activating mutations have been described to cause a rare form of MODY in adolescence or young adulthood (MODY 12), usually responsive to sulfonylurea treatment [17]. At the opposite end, it is noteworthy that inactivating (loss-of-function) mutations in these two genes are important causes of persistent neonatal hypoglycemia due to congenital hyperinsulinism [18]. Permanent NDM can be also caused by heterozygous mutations in the *INS* gene, which encodes for preproinsulin [19]. Such patients will require insulin therapy for life, since *INS* gene mutations lead to misfolding of the insulin protein, its accumulation in various cellular compartments, and, ultimately, beta cell death [20,21]. Homozygous *GCK* mutations have also been described in cases of permanent NDM [22], along with mutations in several other genes.

The wide range of genetic defects involved in the pathogenesis of NDM leads to significant phenotypic variability; patients may, therefore, present clinically with hyperglycemia of varying severity, ranging from incidentally diagnosed asymptomatic hyperglycemia to DKA and severe dehydration [8,13]. Clinically, such neonates are being born small for gestational age due to lack of adequate intrauterine insulin production and, therefore, reduced growth effect, failure to thrive in infants that are not adequately treated, and the typical osmotic features of hyperglycemia such as polyuria and polydipsia. If DKA ensues, the infant presents with lethargy, irritability, tachypnea, and signs of hypovolemia, such as sunken eyes and fontanels. Interestingly, infants with mutations in either *KCNJ11* or *ABCC8* will have DKA in 30–75% of cases; those with *INS* mutations will present with DKA in up to 30%, while transient NDM due to overexpression of *6q24* is not typically associated with DKA [24]. In some cases, especially in NDM due to mutations in *NEUROG3* and *PDX1* genes [25], pancreatic exocrine insufficiency with resultant malabsorptive diarrhea can be identified even in the absence of agenesis or hypoplasia of the pancreas.

In addition, NDM may present with various extra-pancreatic clinical features due to the expression of the affected genes in various tissues and organs throughout the body. Mutations in specific genes can be suspected, depending on specific findings. For example, *WFS1* and *PAX6* gene mutations are usually associated with ophthalmic abnormalities, *WFS1* and *SLC19A2* with deafness, *GLIS3* with hypothyroidism, *GATA4* and *GATA6* with cardiac abnormalities, *EIF2A* and *SLC2A2* with hepatic dysfunction, *HNF1B* with kidney disease, *IPEX*, *STAT1*, *STAT3*, and *LRBA* with immune dysfunction, *EIF2A* with skeletal abnormalities, and *KCNJ11*, *NEUROD1*, *PTF1A*, *IER3IP1*, and *CNOT1* with neurological abnormalities [13,26,27].

In up to 10% of NDM cases, diabetes presents as part of various syndromes diagnosed during infancy. The most common of these is Wolcott–Rallison syndrome (OMIM#226980), due to mutations in *EIF2A*, a gene important in the endoplasmic reticulum function [28]. This syndrome is transmitted in an autosomal recessive manner, and apart from diabetes, other characteristic features include skeletal dysplasia and hepatic dysfunction [29]. In IPEX syndrome (immune dysregulation, polyendocrinopathy, enteropathy X-linked syndrome) (OMIM #304790), mutations in the *FOXP3* gene which encodes for a transcription factor lead to a rare genetic autoimmune disease which manifests with NDM, enteropathy, and polyendocrinopathy [30]. Another syndromic form of NDM is DEND syndrome (developmental delay, epilepsy, and neonatal diabetes) (OMIM#618856) caused by severe mutations in the *KCNJ11* gene, leading to NDM together with epilepsy, severe developmental delay, muscle weakness, and dysmorphic features [31]. Interestingly, early initiation of treatment with sulfonylurea in such patients has been associated with improved neurodevelopmental outcomes [32]. Another rare syndrome is Thiamine-responsive megaloblastic anemia or Rogers syndrome (OMIM#249270), an autosomal recessive disorder caused by mutations in the *SLC19A2* gene. It is characterized by megaloblastic anemia, progressive sensorineural hearing loss, and diabetes with age of onset from infancy to adolescence [33]. The so-called Mitchell–Riley syndrome (OMIM #615710), another rare syndromic form of NDM, is a condition caused by homozygous mutation in the *RFX6* gene and is characterized by pancreatic hypoplasia leading to permanent NDM, duodenal and jejunal atresia, and gall bladder agenesis [34]. Heterozygous mutations in this gene have recently been associated with a rare form of MODY appearing in adolescence [35].

Regarding treatment of NDM patients, in case they appear with DKA and severe dehydration, the appropriate protocol must be implemented with rehydration through intravenous administration of fluids, electrolytes correction, and insulin administration, preferably through an insulin pump. After the infant is stable and has started oral feedings, subcutaneous insulin administration can be initiated either through multiple daily injections or an insulin pump. Subsequently, two possible therapeutic approaches can be followed depending on the specific genetic cause (Figure 2). The first pertains to 50–60% of all infants with permanent NDM that will require subcutaneous insulin treatment for life. Patients with mutations of the *INS* gene account for up to 20% of such cases [9,19], in addition to almost 10% of patients with *KCNJ11* mutations who must equally be treated with insulin since they are not responsive to sulfonylureas [36]. NDM cases as part of genetic syndromes also require lifelong insulin therapy. The second therapeutic approach for NDM includes patients who are responsive to treatment with oral sulfonylureas at high doses (e.g., up to 2.5 mg/kg/day of glibenclamide [glyburide]). These cases represent up to 40% of the total NDM cases and are usually caused by heterozygous activating mutations in the *KCNJ11* or *ABCC8* genes, resulting in either permanent (more severe mutations) or transient (milder mutations) DM [36,37]. The basis of sulfonylurea treatment in such cases is that it reverses the continuously open K_ATP_ channels, thus facilitating beta cell membrane depolarization and subsequent insulin release.

Diagnosing monogenic NDM is relatively easy because, in the absence of syndromic clinical features, the only alternative diagnosis is T1D, which is exceedingly rare before 6 months of age. In a large, international cohort study of 1020 patients, genetic testing of infants younger than 6 months identified a form of monogenic NDM in 82% of cases [9]. It is, therefore, recommended that every patient presenting with DM within the first 6 months of life should be genetically tested for NDM. Some studies have shown that genetic testing can be cost-effective up to the age of 9 months, but this cut-off might further increase in the future as more data emerge [5].

### 3.2. Maturity-Onset Diabetes of the Young (MODY)

#### 3.2.1. Case Scenario 2

A 10-year-old pre-pubertal boy was referred for evaluation of obesity and a recently found elevated fasting glucose level during a routine blood test. There was no history of polyuria, polydipsia, or recent weight loss. The child had no significant past medical history but had excessive obesity and a positive family history of diabetes. More specifically, his mother has a long-standing history of being overweight and impaired fasting glucose, and his father is overweight and receives medication for hypertension and dyslipidemia. In addition, the maternal grandfather has normal weight and has had diabetes for the past 40 years. He has received various oral antidiabetic agents without any improvement in his glycated hemoglobin (HbA1c). On physical examination, the boy’s height was 140 cm (50th percentile), weight was 39 kg (90th percentile), and BMI was 20 kg/m^2^ (95th percentile). Blood pressure and pulse were normal, the thyroid gland was not enlarged, and the boy was prepubertal. No skin lesions or hyperpigmentation were identified. The rest of the examination was unremarkable. First screening laboratory tests showed the following: fasting plasma glucose: 128 mg/dL (NR: 70–99), insulin: 9 μIU/mL (NR: 5–15), C-peptide: 1.6 ng/mL (NR: 0.9–1.8), HbA1c: 6.3% (NR < 5.7), pH: 7.36 (NR: 7.35–7.45), bicarbonate: 23 mEq/L (NR: 22–26), and blood ketones: 0.4 mmol/L (NR < 0.5). Further results included fasting total cholesterol: 185 mg/dL (NR: <170), serum triglycerides: 198 mg/dL (NR < 130), HDL-cholesterol: 38 mg/dL (NR > 45), LDL-cholesterol: 107 mg/dL (NR: 110–129), AST: 42 IU/I (NR: 10–40), ALT: 52 (NR: 10–40), magnesium: 1.8 mg/dL (NR: 1.7–2.1), and normal urine analysis. An oral glucose tolerance test (OGTT) showed plasma glucose at 2 h: 145 mg/dL and insulin level: 53 μIU/mL. All four anti-pancreatic autoantibodies were measured as negative. In addition, no features suggestive of secondary diabetes (drugs, acute illness, Cushing’s syndrome, pheochromocytoma, etc.) or syndromic diabetes were identified either from the personal history or from the clinical examination.

The child described in imaginary case scenario 2 is not severely obese, has a HbA1c in the prediabetic range, and fasting glucose in the diabetic range but without the clinical characteristics typical of diabetes, i.e., polyuria, polydipsia, or recent weight loss. In addition, his OGTT glucose values are affected, while there is no evidence of glucosuria, acidosis, or ketonuria. Both plasma insulin as well as serum C-peptide levels are not low, although inadequate for the respective glucose levels, and autoantibodies related to autoimmune type 1 diabetes are negative. During OGTT, both fasting and stimulated insulin levels are within the normal range, although, as already stated above, inadequately low for the respective glucose levels, and there are no clinical features of severe insulin resistance such as acanthosis nigricans. These findings, taken together with the extensive maternal family history of diabetes, suggest that this child may have a form of MODY rather than T1D or T2D, most probably GCK-MODY.

#### 3.2.2. MODY Types

To date, mutations in various genes have been linked to 14 distinct subtypes of MODY (Table 1). Each of these genes is responsible for a specific step in normal pancreatic beta cell formation and function. Each of the 14 MODY types differs in age of onset and severity of hyperglycemia and in extra-pancreatic manifestations, risk of complications, and need for treatment. It is noteworthy that even mutations in the same gene may lead to variable clinical features and disease severity among members of the same family. The reported prevalence of each MODY subtype differs from country to country due to differences in screening recommendations and financial limitations that may confine extensive genetic testing. However, mutations in *HNF1A*, *GCK*, and *HNF4A* are the most common causes of MODY, accounting collectively for 85–90% of all cases [26]. For example, in a large study from Exeter, UK, the prevalence rates for each of these MODY types were 52%, 32%, and 10%, respectively [38]. It is noteworthy that in this study, the authors concluded that assuming a minimal prevalence of 108 cases per million in the UK population, >80% of MODY cases are not diagnosed via molecular testing. A detailed description of the more common MODY types along with a briefer presentation of the rarer types follows.

#### 3.2.3. HNF1A MODY

HNF1A-MODY (also known as MODY3) is the most common MODY type worldwide with a reported prevalence among all MODY types between 15% and 52% [38,39,40,41]. In pediatric populations, it is the second most common type after GCK-MODY, since the latter is usually diagnosed early during childhood [42]. The *HNF1A* gene is located on chromosome 12q24.2 and encodes for a transcription factor expressed in the adult pancreas, liver, gut, and kidney, the so-called hepatocyte nuclear factor 1 alpha (HNF1A). This factor is important for cell growth, differentiation, and function of beta cells among others and directly regulates the expression of sodium–glucose cotransporter 1 (SGLT1) [43]. Various types of gene variants have been described, for example, frame shift, splicing, or missense and nonsense mutations, as well as duplications, deletions, insertions, or partial and whole-gene deletions. In addition, mutations in different exons of the gene can be identified, influencing its activity to varying degrees [44]. These different gene mutations can affect the age of disease onset, which ranges from early adolescence to late adulthood, but typically the disease presents before 25 years of age [45]. Interestingly, maternal gene inheritance and exposure to diabetes in utero has been linked with an earlier age of diabetes onset [46]. In patients with heterozygous *HNF1A* mutations, insulin secretion in response to glucose is defective and gradually worsens due to a progressive beta cell dysfunction.

Clinically, patients with HNF1A-MODY frequently present with gradually progressive hyperglycemia but usually without DKA, on one hand, or obesity and signs of insulin resistance on the other. Glycosuria may be noted before the identification of overt hyperglycemia, which characteristically appears with a large incremental rise in glucose levels between fasting and 2 h during OGTT. Negative T1D-related autoantibodies in addition to relative preservation of serum C-peptide levels can help in distinguishing from T1D. Other laboratory findings include higher serum ghrelin and HDL cholesterol and lower serum hsCRP as opposed to patients with T1D or T2D [47,48]. A progressive insulinopenia can also be noted with time. A family history of hyperglycemia, consistent with an autosomal dominant inheritance pattern, can be identified.

In the long-term, the risk of microvascular complications is directly associated with glycemic control [49]. Therefore, the correct and timely identification of patients with HNF1A-MODY and the initiation of treatment with a sulfonylurea, which is the first-line treatment in such patients, is of paramount importance. Once the correct diagnosis is made, if the patient was on insulin treatment, insulin can then be stopped and switched to sulfonylurea, e.g., glibenclamide or gliclazide at the lowest dose initially with a gradual up-titration until optimal glycemic control is achieved [50]. Two other classes of anti-diabetic drugs, namely meglitinides [51] and GLP-1 receptor agonists [52], have been tried in patients with HNAF1A-MODY with promising results and with a lower risk of hypoglycemia compared to sulfonylureas. In cases of deterioration of glycemic control over time due to delayed diagnosis or possibly weight gain, change of sulfonylurea treatment to basal insulin, GLP-1 agonists, or metformin has been suggested [50]. SGLT-2 inhibitors have also been tried but the therapeutic benefits are unclear and there is an increased risk of euglycemic ketoacidosis [53].

#### 3.2.4. GCK MODY

GCK MODY (previously known as MODY 2), is the most common form of MODY in childhood, accounting for 88–95% of pediatric cases [41,42], and the second most common form overall. The *GCK* gene is located on chromosome 7p15–p13 and encodes for glucokinase, an enzyme responsible for catalyzing the first reaction in glycolysis which converts glucose to glucose-6-phosphate and modulates insulin secretion in response to intracellular glucose level changes [54]. GCK MODY results from inactivating heterozygous mutations in the *GCK* gene while activating mutations lead to constitutive hyperinsulinism and hypoglycemia. Compound heterozygous or homozygous inactivating mutations result in permanent NDM [55,56]. To date, more than 600 inactivating mutations in all 10 exons of *GCK* have been reported resulting in GCK MODY of variable severity. The resultant GCK malfunction leads to decreased beta cell sensitivity to elevated glucose levels which results in a higher set point for glucose-stimulated insulin secretion [57]. Apart from the beta cells of the pancreas, *GCK* is also expressed in other organs such as the liver and the brain, possibly playing a role in glucose sensing in these tissues [54].

Clinically, most patients are discovered incidentally during routine blood screening. They usually manifest mild fasting non-progressive hyperglycemia, which can be identified since birth and is remarkably stable as the patient grows. Fasting blood glucose levels rarely exceed 125 mg/dL, and, usually, there is mild impaired glucose tolerance during OGTT [58]. Consequently, there is mild A1c elevation ranging between 5.8 and 7.6% (40–60 mmol/mol) in younger patients, and somewhat higher in those over 40 years of age [59]. What is characteristic of GCK MODY patients is that their lifelong risk of macrovascular and microvascular complications is equivalent to that of healthy controls, despite their persistent mild hyperglycemia [60].

Since patients with CGK MODY present with lifelong mild stable hyperglycemia, and they do not have increased risk of lifelong complications, there is a consensus that pharmacological treatment is not required. Indeed, glucose-lowering agents or insulin use because of misdiagnosis of the type of diabetes has led to hypoglycemia and other adverse effects in more than a third of these patients [61]. The only exception is if T2D or T1D coexists and during pregnancy, where, depending on fetal genotype, a different therapeutic approach is required for the optimal outcome of both the fetus and the mother [62]. Interestingly, in GCK MODY pregnancies, when the fetus inherits a maternal pathogenic *GCK* variant, impaired sensing of elevated maternal glucose “protects” it from increased insulin secretion and birthweight is unaffected. On the other hand, when the pathogenic *GCK* variant is not inherited, insulin secretion increases in response to maternal hyperglycemia, increasing the risk for macrosomia; in such cases, insulin treatment of maternal hyperglycemia is recommended. Although fetal genotype can usually be postulated based on fetal abdominal circumference as measured via ultrasound, cell-free DNA collection from maternal blood for fetal *GCK* genotype determination has recently been developed with very promising results [63].

#### 3.2.5. HNF4A MODY

HNF4A MODY (previously MODY1) is diagnosed less commonly, accounting for 5–10% of all MODY cases [38]. It is caused by mutations in the *HNF4A* gene which is located on chromosome 20q13.2 and encodes for the hepatocyte nuclear factor 4 alpha (HNF4A), a protein that belongs to a family of ligand-activated transcription factors. The *HNF4A* gene is expressed in various organs including the pancreas, the liver, the intestine, and the kidney, encoding for nine different isoforms of the HNF4A protein [64]. In the pancreas, the HNF4A protein regulates, among others, the expression of the *HNF1A* gene, thus playing a role in the regulation of glucose-mediated insulin production [65]. More than 400 mutations of the *HNF4A* gene have been described thus far: mainly missense mutations, but also splice-site and frameshift mutations and gene deletions. Just like *HNF1A* mutations, the defect location can affect the age of onset and the severity of hyperglycemia but without a definite genotype–phenotype correlation [66].

Clinical presentation of patients with HNF4A MODY is similar to that of patients with HNF1A MODY, namely hyperglycemia appearing in adolescence or early adulthood (before 25 years of age), with gradual deterioration due to progressive beta cell dysfunction. A specific feature of this MODY type is that up to 50% of patients develop hyperinsulinemia in utero which leads to fetal macrosomia and hypoglycemia at birth. After birth, hyperinsulinemia subsides, and the patient is euglycemic in childhood but with gradual beta cell dysfunction leading to overt diabetes [67,68].

Similar to HNF1A MODY, the rate of diabetes complications in patients with HNF4A MODY is directly related to glycemic control and is comparable to that of patients with T1D or T2D. Therefore, early and accurate diagnosis of the disease is important to timely initiate sulfonylurea treatment, just like in HNF1A MODY; although, most patients will eventually require insulin therapy [69]. The literature on other therapeutic approaches is much scarcer than in HNF1A MODY [50].

#### 3.2.6. HNF1B MODY

HNF1B MODY (previously MODY5) accounts for approximately 2–6% of all MODY cases and is the fourth most common type [38]. It is caused by heterozygous mutations of the *HNF1B* gene, which is located on chromosome 17q12, and which encodes for the hepatocyte nuclear factor 1 beta (HNF1beta) protein (also known as Transcription Factor 2, TCF2). This protein belongs to the homeobox-containing transcription factor family and plays a significant role in the development of several tissues and organs including the pancreas, lung, gut, kidney, and liver [70]. More than 50 *HNF1B* mutations have been described that lead to the HNF1B MODY phenotype. Such mutations can appear de novo and should be suspected in patients with diabetes and renal cysts or other renal abnormalities even in the absence of a relevant family history.

Clinical presentation is highly variable and, apart from pancreatic and renal dysfunction, includes abnormal hepatic function, genital tract malformations, and increased cancer risk, in various combinations [70,71]. In addition, hyperuricemia, abnormal liver function tests, and renal magnesium wasting with consequent hypomagnesemia can be identified [72]. In childhood, patients may sometimes present with isolated renal cysts without overt diabetes [73]. In more than half of HNF1B MODY cases, large genomic rearrangements or whole-gene deletions can be identified, resulting in the so-called 17q12 deletion syndrome. In this case, neurodevelopmental and autistic-spectrum disorders may coexist [74].

Regarding management of patients with HNF1B MODY, sulfonylureas, GLP-1 analogs, and meglitinides have been tried with some success shortly after diagnosis [50,75]. Nevertheless, because of progressive beta cell function deterioration, insulin remains the mainstay of diabetes treatment for most of these patients [50]. Concerning kidney function, some patients maintain normal renal function, but others develop chronic kidney disease and require dialysis or transplantation at some point of their lives [76].

#### 3.2.7. Rarer Types of MODY

PDX1/IPF1 MODY, previously known as MODY4, is caused by mutations in the *PDX1*/*IPF1* gene which is located on 13q12.2 and is important for the formation of pancreatic beta cells and the gut, especially the duodenum. In addition, the PDX1 protein is involved in the expression of multiple genes, including those regulating insulin, somatostatin, and glucokinase production. It has been shown in mouse models that homozygous deletion of this gene leads to pancreatic agenesis [77], while heterozygous mutations, such as in patients with PDX1/IPF1 MODY, lead to impaired insulin production and hyperglycemia [78]. Treatment is variable according to the severity of clinical presentation and includes diet alone, hypoglycemic agents, and/or the addition of insulin.

Mutations in *ABCC8*, *KCNJ11*, and *INS* genes are mostly associated with neonatal diabetes, as described above in detail. Rarely, they can appear as MODY and, collectively, they are responsible for <1% of all MODY cases. Clinically, such patients usually present with diabetes like patients with HNF1A or HNF4A MODY [19,79,80].

#### 3.2.8. Distinguishing MODY from Other Types of Diabetes

**Figure 3:**
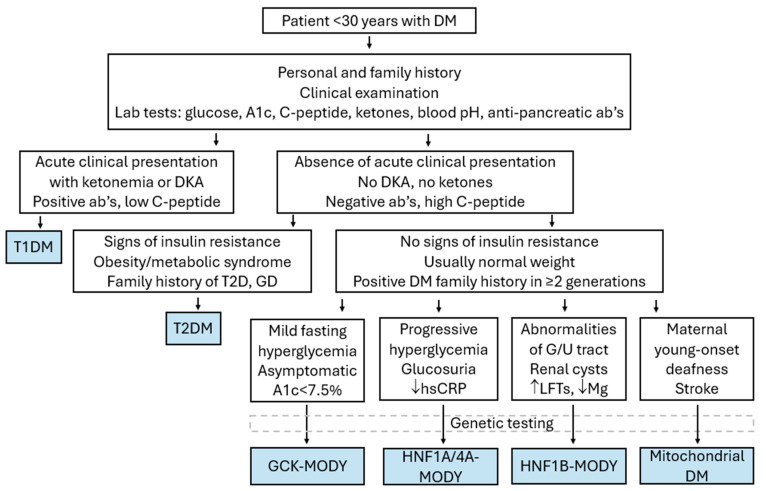
A practical clinical algorithm for the assessment of a young patient with diabetes. A1c: glycated hemoglobin, Ab’s: antibodies, DKA: diabetic ketoacidosis, DM: diabetes mellitus, GCK: glucokinase, GD: gestational diabetes, G/U: genito-urinary, HNF: hepatocyte nuclear factor, hsCRP: high sensitivity c-reactive protein, LFT: liver function test, Mg: magnesium, T1D: type 1 diabetes mellitus, T2D: type 2 diabetes mellitus.

Since management of patients with MODY differs substantially from that of patients with T1D or T2D, it is important to timely and accurately establish the diagnosis of monogenic diabetes in such cases (Table 2, Figure 3). Originally, MODY was defined by the following criteria: onset before 25–35 years of age, lack of insulin dependence (evident by C-peptide levels or type of treatment), lack of obesity, signs of insulin resistance, and autosomal dominant inheritance over multiple generations [1]. However, accurately diagnosing monogenic diabetes is complicated by the fact that different MODY subtypes have variable clinical features that may overlap with other types of diabetes. Genetic testing is the mainstay of diagnosis, but financial restrictions in most countries preclude universal genetic testing of all patients with diabetes [81]. In addition, such an approach would probably lead to a high number of “variants of uncertain significance” (VUS), which would be difficult to interpret clinically [5]. No screening approach has been proven sensitive enough to detect accurately all patients ultimately proven to have MODY or specific enough to make sure that genetic testing will be carried out only on patients with MODY. Therefore, clinical judgment as to who of all patients with diabetes must be genetically tested for MODY is important and should be based upon a combination of clinical findings and laboratory testing. A useful clinical prediction tool developed by the University of Exeter in the UK is the MODY probability calculator, available online: www.diabetesgenes.org/exeter-diabetes-app/ (accessed on 15 August 2024) and can help clinicians estimate the probability of a patient having MODY, based on simple clinical and laboratory criteria [82].

For the pediatric cases with diabetes, the main alternative diagnosis is T1D. Testing for multiple T1D-related autoantibodies, such as anti-GAD65, anti-insulin, anti-IA2, and anti-ZnT8 antibodies, is important since these are increased in 85–90% of patients with T1D. When two or more of these are positive, a diagnosis of T1D can be established, thus precluding the need for genetic testing [83,84]. However, 10–15% of pediatric patients with diabetes have the so-called type 1B diabetes, which is autoimmune diabetes with negative antibodies at the time of diagnosis. This percentage decreases with time and with repeat testing [85]. Data from the multicenter SEARCH for Diabetes in Youth study in the U.S. demonstrated that 85–92% of children with diabetes and negative autoantibodies have T1D, 7–15% have MODY, and the rest have T2D, with varying percentages depending on the population studied [39]. Conversely, one must keep in mind that a small percentage of individuals without T1D (up to 2%) can have positive autoantibodies, usually anti-GAD65 [86]. Apart from antibodies, other laboratory findings that would increase the possibility of MODY are a relatively low HbA1c < 7.5% and the absence of DKA at the time of diagnosis, along with preserved C-peptide production, suggesting persistent beta cell function and insulin secretion. Of course, one must keep in mind that T1D cannot be excluded based on C-peptide alone, since serum C-peptide levels may be measurable at an early stage following diagnosis or in the so-called “honeymoon period” [87]. Further, a strong family history of diabetes for more than two consecutive generations with an autosomal dominant pattern of inheritance is suggestive of MODY [84].

On the other hand, cases of T2D are increasingly identified in adolescents, especially those belonging to high-risk racial/ethnic groups. In such patients, obesity, increased insulin resistance with acanthosis nigricans, and hyperinsulinemia on OGTT, as well as a positive family history of T2D and/or gestational diabetes, could help differentiate between T2D and MODY [88]. Still, just like the case scenario 2 described at the beginning of the chapter, it is not always easy to differentiate between the two clinical entities, since, in many cases, adult patients with MODY are treated as having T2D, thus making proper diagnosis of their offspring rather challenging.

### 3.3. Syndromic Monogenic Diabetes Mellitus

#### 3.3.1. Case Scenario 3

A 16-year-old adolescent male was admitted to the Hospital following an episode of seizures followed by aphasia. Blood glucose levels were consistently greater than 200 mg/dL since admission. During the month prior to his admission, the patient complained of fatigue, polydipsia, and polyuria. There was a past medical history of recurrent migraine headaches and sensorineural deafness treated with hearing aids. Regarding his family history, a maternal uncle had insulin-treated diabetes, recurrent strokes starting at the age of 25, and hearing loss since childhood. He died from congestive heart failure at 55 years of age. On physical examination, the boy’s height was 164 cm (15th percentile), weight 50 kg (50th percentile), and BMI 18.6 kg/m^2^ (normal). His Tanner stage was GIII, PIII, A++.

The patient in this imaginary case scenario most probably suffers from MELAS (mitochondrial myopathy, encephalopathy, lactic acidosis, and stroke-like episodes) syndrome.

#### 3.3.2. Diabetes Mellitus as Part of a Syndrome

Several syndromes can be associated with monogenic diabetes mellitus that manifests in adolescence or early adulthood, including MELAS, MIDD syndrome (maternally inherited diabetes and deafness), Wolfram syndrome, Kearns–Sayre syndrome, and Pearson syndrome.

MELAS syndrome (OMIM #540000) is a mitochondrial disorder that affects various body systems, especially the nervous system and the muscles, which are tissues with high energy demands [89]. It is caused by mutations in the mitochondrial DNA, with the most common being the m.3243A > G point mutation in the *MT-TL1* gene which encodes for the mitochondrial tRNA (Leu(UUR)). Since it is a mitochondrial disease, it is maternally inherited. In fact, various syndromes including MIDD (maternally inherited diabetes-deafness syndrome, OMIM #520000), MELAS, and CPEO (chronic progressive external ophthalmoplegia) are due to the same m.3243A > G mutation. There is significant phenotypic overlap, and DM is a common feature of all three syndromes. MELAS syndrome typically presents in adolescence or early adulthood, with a variable clinical phenotype. Stroke-like episodes leading to recurrent headaches, epilepsy, and neurological impairments, muscle weakness, exercise intolerance, and progressive sensorineural hearing loss, as well as lactic acidosis that leads to nausea, vomiting, fatigue, and abdominal pain, are typical features of the disease. DM appears in approximately 20–40% of patients with MELAS syndrome. Mitochondrial DM is estimated to be the cause of ~1% of the total DM population and more than 85% of these cases are caused by the m.3234A > G pathogenic variant. Therapeutically, insulin, incretin analogs, and/or oral antidiabetic medications can be used, apart from metformin because of the increased risk of lactic acidosis. The pathophysiology of mitochondrial diabetes is complex, as apart from impaired beta cell function, some patients may manifest features of T2D such as insulin resistance and acanthosis nigricans [89,90,91].

Wolfram syndrome 1 is a very rare autosomal recessive disease caused by mutations in *WFS1* and *WFS2* genes that produce wolframin, which is a protein important for the function of endoplasmic reticulum. Main clinical features of the syndrome include diabetes insipidus (DI), early-onset non-autoimmune insulin-dependent diabetes mellitus (DM), optic atrophy (OA), and deafness (D), hence the earlier used acronym DIDMOAD. In addition, urinary tract, neurological, and psychiatric abnormalities can be present. Patients with a similar disease named Wolfram syndrome 2 also appear with DM, hearing loss, and optic atrophy but the genetic cause is different, involving mutations in the *CISD2* gene [92].

Several other syndromes, such as Kearns–Sayre syndrome, Pearson syndrome, and Alström syndrome, can include hyperglycemia and diabetes as a secondary clinical manifestation. However, DM is present in only a small fraction of these patients, and the diagnosis is usually established mainly on the basis of other clinical features that are characteristic for each syndrome. A detailed description of these syndromes is beyond the scope of this review.

### 3.4. Limitations

The main limitations of the current review are inherent to its narrative rather than systematic nature. Nevertheless, we aimed to summarize the latest data on monogenic defects affecting beta cell function, using examples of typical clinical scenarios and discussing the differential diagnosis, genetic confirmation, and optimal management with the practicing clinician in mind.

## 4. Conclusive Remarks

The progress in understanding genetic defects leading to monogenic diabetes has been substantial in recent years. This progress has increased the accuracy of diagnosing even the rarer subtypes, has improved our knowledge on the most probable clinical course of each patient, and has helped in deciding the best treatment based on a precision medicine approach. Nevertheless, many aspects of these elusive disorders remain unknown, making further research imperative. In addition, until more cost-effective and more accurate genetic tests allow for universal screening of all patients with diabetes, clinical judgement based on knowledge and experience is essential in order to identify those patients who will most probably test positive for a genetic defect and will benefit the most from such a diagnosis and the recommended treatment.

## Figures and Tables

**Table 1:** MODY type associated genes, earlier nomenclature, genetic locus, and associated defects of beta cell development or function.

Gene	Previous Name	Chromosome	Associated Beta Cell Dysfunction
*HNF1A*	MODY 3	12q24.2	Impaired glucose-mediated insulin secretion
*GCK*	MODY 2	7p15-p13	Normal beta cell secretion capacity but with higher glucose threshold for insulin secretion
*HNF4A*	MODY 1	20q13.2	Impaired insulin secretion with progressive deterioration in beta cell function
*HNF1B*	MODY 5	17q12	Beta cell dysfunction accompanied by pancreatic agenesis or atrophy
*PDX1*	MODY 4	13q12.2	Defective differentiation of beta cells; impaired insulin secretion after glucose load
*NEUROD1*	MODY 6	2q32	Dysfunction of beta cells accompanied by insulinopenia or insulin resistance
*KLF11*	MODY 7	2q25	Insufficient insulin synthesis; impaired glucose-mediated insulin secretion
*CEL*	MODY 8	9q34	Beta cell dysfunction due to protein misfolding leading to its aggregation; pancreatic atrophy
*PAX4*	MODY 9	7q32	Defective maturation of beta cells; impaired glucose-mediated insulin secretion
*INS*	MODY 10	11p15.5	Apoptosis of beta cells; gradual reduction in beta cell mass
*BLK*	MODY 11	8p23	Reduced beta cell mass; defective glucose-mediated insulin secretion
*ABCC8*	MODY 12	11p15.1	Insufficient insulin secretion
*KCNJ11*	MODY 13	11p15	Insufficient insulin secretion
*APPL1*	MODY 14	3p14.3	Decreased beta cell survival; defective glucose-mediated insulin secretion

**Table 2:** Clinical and laboratory differences between MODY diabetes and type 1 and type 2 diabetes in childhood and adolescence.

Parameter	MODY Diabetes	T1D	T2D
Prevalence	Rare, stable	Common, increasing	Rare, increasing
Ethnicity	All	Mainly Caucasian	Mainly minority groups
Inheritance	Autosomal dominant	Multigenic	Multigenic
Family history	Almost 100% positive for MODY	5%-10% positive for T1D	75%-90% positive for T2D
Sex	Male = Female	Male = Female	Male < Female
Age at presentation	Before 25 yr of age	Childhood-adolescence	Adolescence
Body habitus	Various	Usually normal weight	Mostly obese
Acanthosis nigricans	Absent	Rare	Very common
Onset	Insidious	Usually acute, severe	Usually insidious, rarely acute
Onset with ketosis	Rare	Common	5%–10%
Insulin, C-peptide	Detectable	Decreased or absent	Variable
Insulin sensitivity	Normal	Normal	Decreased
HLA-DR3/4 association	None	Strong	None
Pancreatic autoantibodies	Rare	85%–100%	<10%
Insulin dependence	Rare	Permanent	Variable
Associated disorders	Depending on type, exocrine pancreas insufficiency, urogenital malformations, etc.	Autoimmune disorders (e.g., Hashimoto’s thyroiditis, vitiligo, celiac disease)	MetS components (e.g., lipid disorders, hypertension, PCOS, sleep apnea, etc.)

HLA: human leukocyte antigen; MetS: metabolic syndrome; MODY: maturity-onset diabetes of the young; PCOS: polycystic ovary syndrome, T1D: type 1 diabetes mellitus; T2D: type 2 diabetes mellitus.
